# Supplementary material for: The photosystem I assembly apparatus consisting of Ycf3–Y3IP1 and Ycf4 modules
Source: Nat Commun. 2018 Jun 22;9:2439. doi: 10.1038/s41467-018-04823-3 (PMC6015050; doi:10.1038/s41467-018-04823-3)
Supplement: Supplementary file 1 — Supplementary Information [file 41467_2018_4823_MOESM1_ESM.pdf]

Supplementary information

The photosystem I assembly apparatus consisting of Ycf3-Y3IP1 and Ycf4 modules

Nellaepalli et al

Supplementary Figure 1

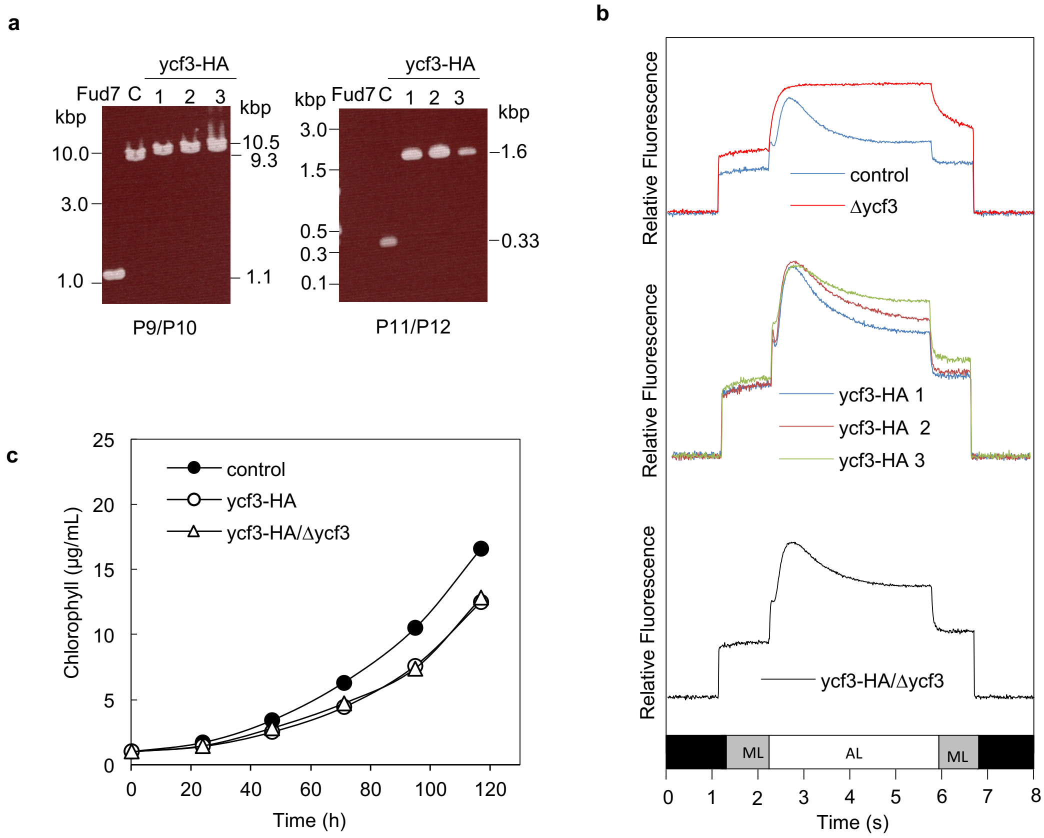

Supplementary Figure 1. Characterization of ycf3-HA and ycf3-HA/Δycf3 mutants. (a) Confirmation of the genotype of the ycf3-HA mutants (clones 1-3), control strain (C), and Fud7. Total cellular DNA was amplified by PCR using P9/P10 primers (left) or P11/P12 primers (right). A 1.1 kbp PCR product using P9/P10 primers from Fud7 shows the deletion around the *psbA* gene while a 9.3 kbp PCR product from control indicates the presence of the *psbA* gene. A 10.5 kbp PCR product from ycf3-HA (clones 1-3) mutants indicates the insertion of the *ycf3-HA* expression cassette. Using P11/P12 primers, no PCR product was amplified from Fud7 while a 0.33 kbp PCR product from control was amplified, indicating the absence and the presence of *psbA* gene, respectively. A 1.6 kbp PCR product from ycf3-HA (clones 1-3) mutants indicates the insertion of the expression cassette. **(b)** Chl *a* fluorescence kinetics measured with a Dual-PAM. Δycf3 mutant showed no PSI activity, whereas ycf3-HA (clones 1-3) and ycf3-HA/Δycf3 mutants showed PSI activity like control strain. ML; measuring light, AL; actinic light. **(c)** The growth of control, ycf3-HA, and ycf3-HA/Δycf3 mutants in liquid HSM media under illumination at 50 μmol photons m^{-2} s^{-1}.

Supplementary Figure 2

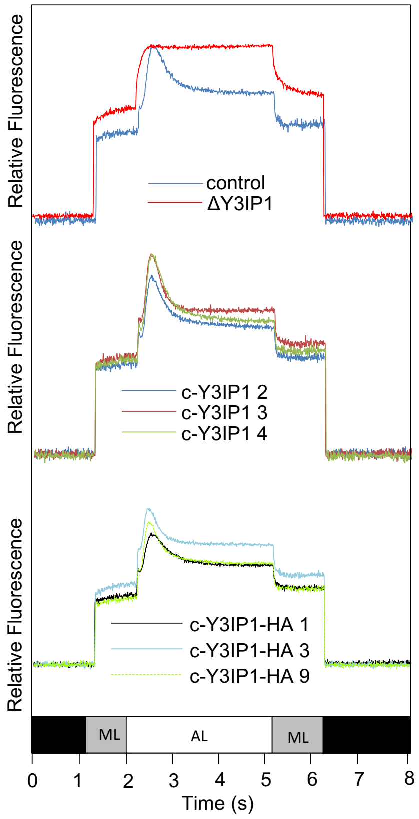

Supplementary Figure 2. Chl *a* fluorescence induction kinetics. Cells of control, ΔY3IP1, c-Y3IP1 (clones 2-4), and c-Y3IP1-HA (clones 1, 3, and 9) were grown in liquid TAP medium to a mid-log growth phase under the light of 5-50 μmol photon m^{-2} s^{-1} and adapted in the dark for 30 min before the measurements with a Dual-PAM fluorescence spectrometry. ΔY3IP1 showed no PSI activity, whereas ycf3-HA and ycf3-HA/Δycf3 mutants showed PSI activity like control strain. ML; measuring light, AL; actinic light.

Supplementary Figure 3

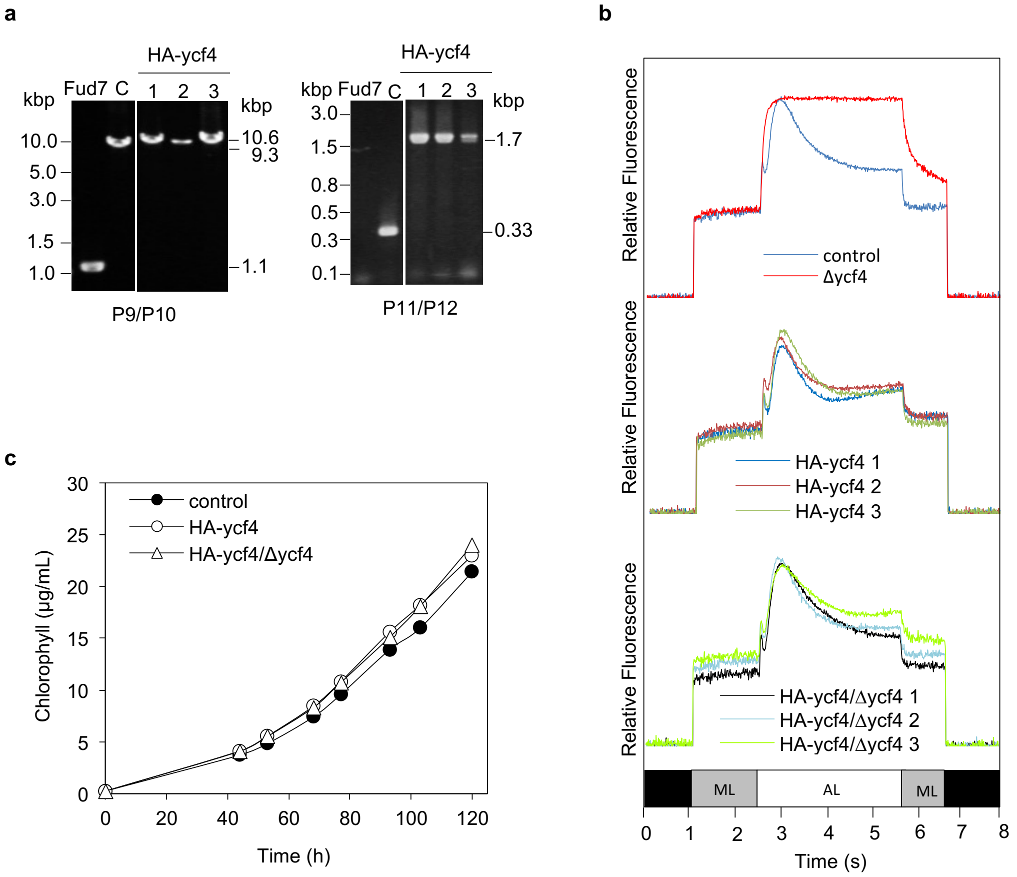

Supplementary Figure 3. Characterization of HA-ycf4 and HA-ycf4/Δycf4 mutants. (a) Confirmation of the genotype of the HA-ycf4 mutants (clones 1-3), control strain (C), and Fud7. Total cellular DNA was amplified by PCR using P9/P10 primers (left) or P11/P12 primers (right). A 1.1 kbp fragment from Fud7 using P9/P10 primers shows the deletion around the *psbA* gene while a 9.3 kbp fragment from control indicates the presence of the *psbA* gene. A 10.6 kbp PCR product from HA-ycf4 (clones 1-3) mutants indicate the insertion of the expression cassette. A 1.7 kbp fragment obtained using P11/P12 primers confirmed the insertion of the expression cassette in the HA-ycf4 (clones 1-3) mutants. **(b)** Chl *a* fluorescence kinetics measured with a Dual-PAM. Δycf4 mutant showed no PSI activity, whereas HA-ycf4 (clones 1-3) and HA-ycf4/Δycf4 (clones 1-3) mutants showed PSI activity like control strain. ML; measuring light, AL; actinic light. **(c)** The growth of control, HA-ycf4, and HA-ycf4/Δycf4 mutants in liquid HSM media at 50 μmol photons m^{-2} s^{-1}.

Supplementary Figure 4

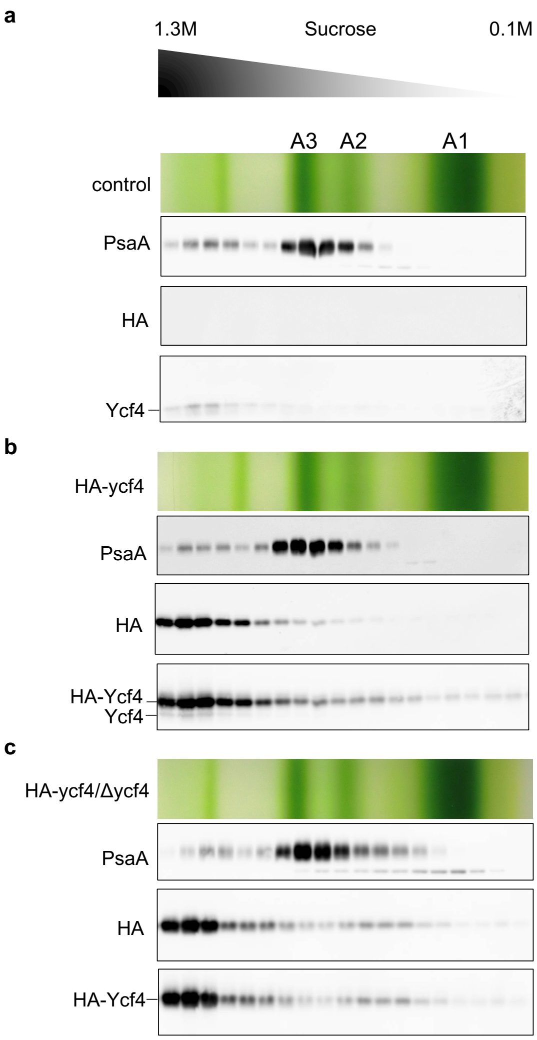

Supplementary Figure 4. HA-Ycf4 is part of a large complex. Thylakoid membranes were solubilized with 1% β-DM and separated by SDG ultracentrifugation. **(a)** control**, (b)** HA-ycf4, **(c)** HA-ycf4/Δycf4. Ycf4 and HA-Ycf4 from the three strains were separated near the bottom of the gradient, indicating HA-Ycf4, as well as Ycf4, are part of a large complex.

Supplementary Figure 5

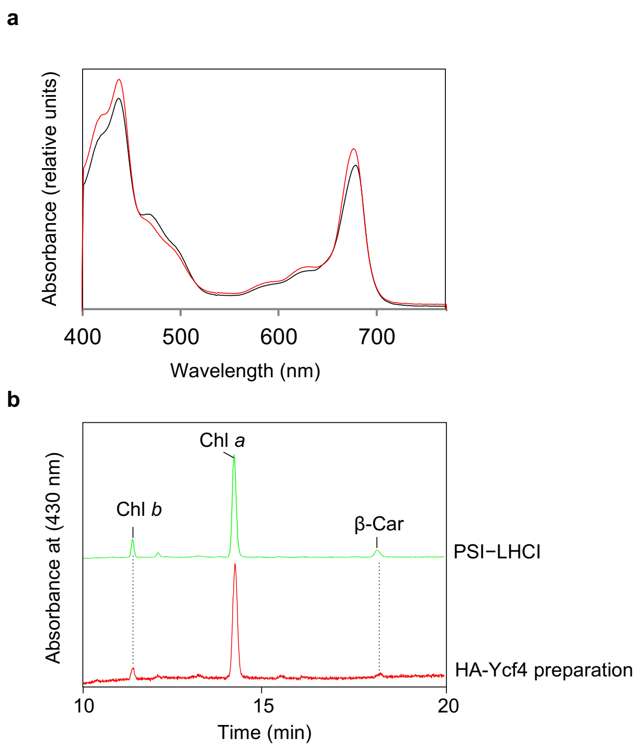

Supplementary Figure 5. Pigment analysis in the affinity-purified HA-Ycf4 preparation. (a) Absorption spectra of the HA-Ycf4 (red line) and PSI–LHCI preparations (black line). (**b)** Pigments extracted from the HA-Ycf4 and PSI–LHCI preparations were separated and identified by HPLC. Chl *a*/*b* ratio was estimated as 7.9 and 4.7 in HA-Ycf4 and PSI−LHCI preparations, respectively.

Supplementary Figure 6

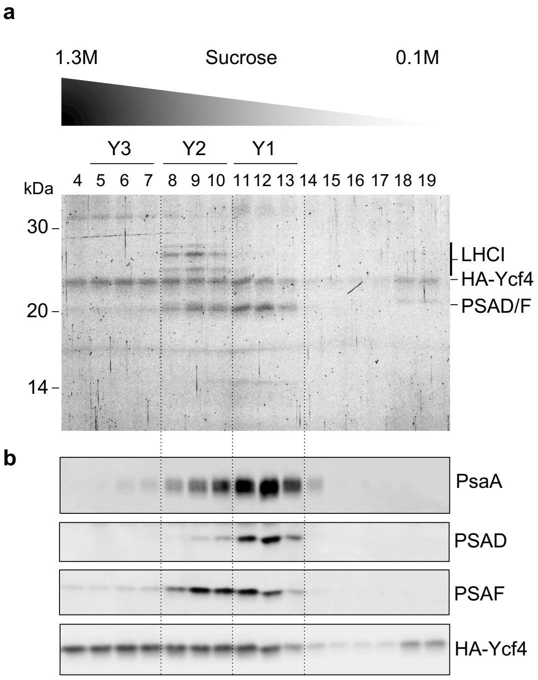

Supplementary Figure 6. Separation of the purified HA-Ycf4 by SDG ultracentrifugation. (a) Fractions collected from SDG of HA-Ycf4 preparation (Fig. 5a) were subjected to SDS-PAGE, and the polypeptides were visualized by staining with Flamingo. (**b)** The presence of PSI subunits in Y1 and Y2 was determined by immunoblotting using anti-PsaA, PSAD/F, and Ycf4 antibodies. HA-Ycf4 was fractionated broadly in Y1, Y2, and Y3.

Supplementary Figure 7

Figure 1b

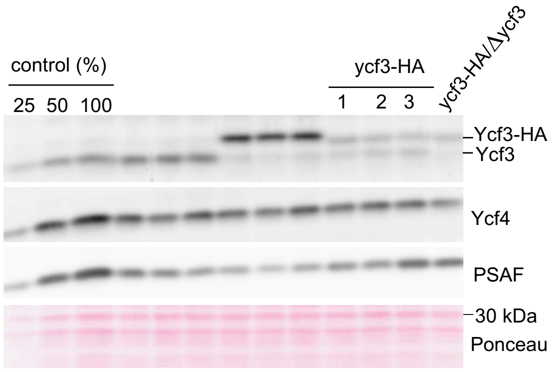

Figure 4b

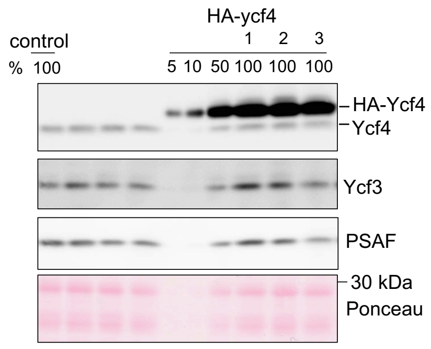

Figure 4c

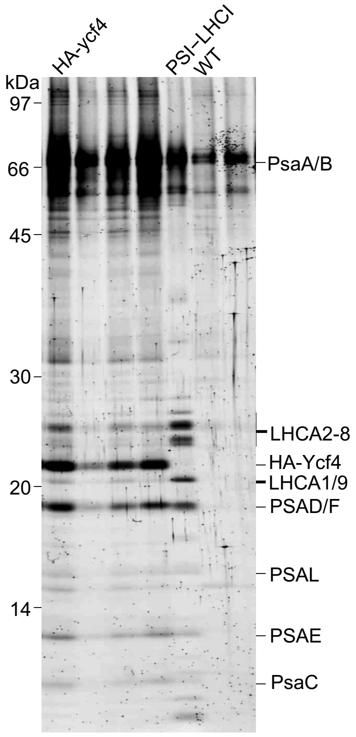

Figure 4d

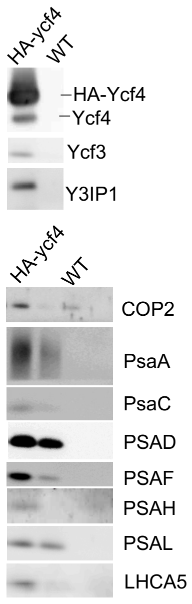

Figure 4f

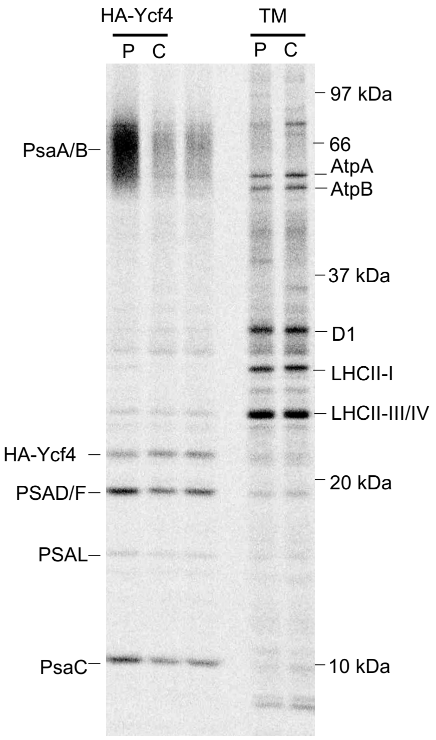

Supplementary Figure 7. Uncropped gel and blot images of the trimmed figures used in this study. Please refer the main text for the figure legends.

Supplementary Table 1

Ycf3-HA preparation

Protein	Accession	Peptide	Position	Z	ΔM	Xcorr
PsaB	NP_958404.1	FSQGLAQDPTTR	9-20	2	0.83	3.73

HA-Ycf4 preparation

Protein	Accession	Peptide	Position	Z	ΔM	Xcorr
PsaA	NP_958375.1	FSNYEAWLSDPTHIK	98-112	2	1.83	5.06
		DYDPTNNYNNLLDR	416-429	2	0.54	4.26
		LLDAGVDPK	227-235	2	0.92	3.21
		EIPLPHDLLLNR	236-247	2	1.66	2.75
PsaB	NP_958404.1	FSQGLAQDPTTR	9-20	2	0.12	3.58
		DKPVALSIVQAR	696-707	2	0.79	3.42
		ALYGFDFLLSSK	471-482	2	0.66	3.37
		TPLANLVYWK	686-695	2	-0.07	2.78

Supplementary Table 1. PsaA and PsaB Polypeptides identified with LC-MS/MS. The stained bands of 66 kDa from Fig. 1c and Fig. 4c for Ycf3-HA and HA-Ycf4 preparations, respectively, were excised and digested with trypsin and the resulting polypeptides were subjected to LC-MS/MS.

Supplementary Table 2

Name	Oligonucleotide sequence
P1	5'-AGAAATCCATGCCAAGAACGCAAAGAAATGATAATTTTATCGAT
P2	5'-CCCCCGCATGCTTAAGTAGCTAAACCTGTTAGACGT
P3	5'-AGAAATCCATGACACAAAATAATATTTTAATTAGACGTTATA
P4	5'-CCCCCGCATGCTTAGGCTTCTAAAGAAACTTGTAAAAAGTT
P5	5'-CTAACAGGTTTAGCTACTTATCCATATGATGTTCCAGATTATGC
P6	5'-TTAAGCATAATCTGGAACATCATATGGATAAGTAGCTAAACCTGTTAGACGT
P7	5'-CCAGATTATGCTACACAAAATAATATTTTAATTAGACGTTATATTATTGTAG
P8	5'-AACATCATATGGATACATGGATTTCTCCTTATAATAACAATTATT
P9	5'-GTTCTGACGCGTTTGTGAGGCTTTATTAAC
P10	5'-TTCTGAAACCGTTCGTGCTGTGCTAGACAG
P11	5'-TTTCGCCATATGTAGACGTTTAATTGCTAC
P12	5'-AGTACCATCAGATATTGCTAGCATGGATAC
P13	5'-CGGCATATGAGCGCGCAGCTCCGAGCTCTTAG
P14	5'-CCTCTAGATTACTTGAGAGAGTTGTAGAACAGGAT
P15	5'-GGGCATATGAGCGCGCAGCTCCGAGCTCTTA
P16	5'-CCTCTAGAATTACTTGAGAGAGTTGTAGAACAGG
P17	5'-GATTACGAATTCGTATTGCGCTCAAGTGCCTGCACGACGA
P18	5'-CACGTCGTAGGGGTACTTGAGAGAGTTGTAGAACAGGATGAGG

Supplementary Table 2. Oligonucleotides used in this study
